# SharpCEEWPServer: A Lightweight Server for the Communication Protocol of China Earthquake Early Warning Systems

**DOI:** 10.3390/s26010262

**Published:** 2026-01-01

**Authors:** Li Li, Jinggang Li, Wei Xiang, Zhumei Liu, Wulin Liao, Lifen Zhang

**Affiliations:** 1 Hubei Key Laboratory of Earthquake Early Warning, Hubei Earthquake Agency, Wuhan 430061, China; ynulili@foxmail.com (L.L.); ljg@hubdzj.gov.cn (J.L.); ailiuzhumei@163.com (Z.L.); lwldyx@163.com (W.L.); 2 Guangxi Earthquake Administration, Nanning 530022, China; 17758641046@163.com

**Keywords:** CSTP, communication protocol, earthquake early warning, lightweight server, temporary seismic networks

## Abstract

Several commercial seismometers now support CSTP, the real-time communication protocol used in the China Earthquake Early Warning System, but there is still no simple, flexible, and low-cost solution to archive CSTP streams or integrate them into existing data processing systems. In this study, we design and implement SharpCEEWPServer, a lightweight, out-of-the-box graphical server that integrates client management, real-time data reception, visualization, and archiving, and can, via RingServer, convert CSTP real-time streams into widely supported SeedLink streams. Hardware compatibility is evaluated using four commercial CSTP-capable instruments, a forwarding chain is built to assess forwarding functionality and reliability, and concurrency performance is tested using simulated networks with different station counts. The stability under network impairment scenarios and the performance of the forwarding system were also analyzed. The results show that the server can reliably receive and forward real-time data streams, and that laptop-class hardware is sufficient to withstand the load imposed by an M7.0 earthquake scenario when receiving real-time streams from 1000 three-component seismometers. However, the current version’s latency performance can only meet the needs of non-early warning networks. Overall, the proposed server significantly lowers the deployment and usage threshold for new CSTP-capable instruments and provides an efficient, low-cost integration solution for temporary networks in earthquake emergency response and seismic arrays.

## 1. Introduction

**Figure 1:**
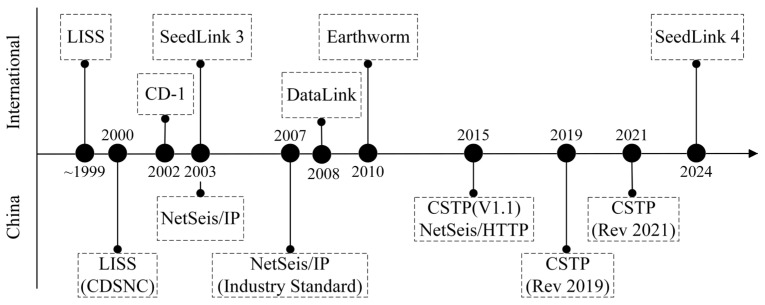
The history of major seismic data communication protocols.

Modern seismic monitoring networks consist of seismometers and data centers, and real-time data transmission between nodes must follow agreed communication protocols. In China, the national seismic monitoring network has been developed as a long-term national program. Over successive stages, the number of stations has steadily increased, communication and data-processing technologies have been systematically upgraded, and the communication protocols in use have been adjusted in response to operational needs. The history of major seismic data communication protocols show in Figure 1.

During the Ninth Five-Year Plan (1996–2000), China’s seismic observation network completed its digital transformation. Forty-seven national reference stations were established, and data were transmitted using one-way satellite asynchronous data streams. In 2000, the China Digital Seismographic Network Center began to provide near real-time data from the Beijing Baijiatuan and Xi’an stations to Chinese and abroad users via the Live Internet Seismic Server (LISS) [1], marking the beginning of network-based operations. However, the LISS protocol only supports one-way transmission and does not allow retransmission of lost data [2], which makes it difficult to meet more complex requirements. It was subsequently superseded internationally by more feature-rich protocols such as SeedLink and CD-1 [3,4,5,6]. In parallel, the China Earthquake Administration (CEA) chose to develop proprietary communication protocols tailored to its own operational characteristics, following a differentiated technical path.

By the Tenth Five-Year Plan (2001–2005), the CEA completed the construction of one national seismic network and 31 regional networks, with a total of 1005 stations, and completed the transition to network-based operation. In this period, the China Seismographic Network Data Specification was formulated, and the NetSeis/IP protocol—a digital network communication protocol designed on the basis of LISS—was incorporated into the specification [7]. Based on this specification, the Guangdong Earthquake Monitoring Center developed the JOPENS5 data processing system for the digital seismic network center using Java 2 Platform, Enterprise Edition. JOPENS5, which includes versions from 5.0 to 5.2, became a core software platform in China’s seismic monitoring system and remains in use today [8]. Although NetSeis/IP includes data-upload functionality, it is supported by very few instruments, so JOPENS5 has to actively accommodate dozens of IP-based and serial communication protocols. This approach not only increases software development complexity but also introduces additional runtime overhead.

Earthquake early warning (EEW) has been deployed and tested as a disaster-mitigation technology in several seismically active regions, including southern California, Japan, Italy, and Turkey [9,10,11,12,13,14,15]. In China, EEW development was driven by the 2008 Wenchuan M8.0 earthquake, which caused severe casualties and economic losses. During the Thirteenth Five-Year Plan (2016–2020), the CEA launched the construction of a nationwide EEW network, carrying out pilot projects in regions such as Beijing, Zhaotong in Yunnan, and the Sichuan–Yunnan border [16,17,18,19], and funding the development of earthquake early warning systems (EEWS). EEWSs are highly sensitive to latency and therefore require large-scale deployments of low-cost accelerometers or even IoT-based terminals [20,21,22,23], together with low-latency communication protocols [24,25]. To meet these requirements, the CEA organized software development institutes and instrument manufacturers to jointly define a dedicated data transmission protocol for seismic intensity meters—referred to as CSTP in early literature (CSTP is the name used in the JOPENS6 documentation. No authoritative source provides an official English expansion of this protocol name)—which was adopted as the standard communication protocol for EEWS [26]. Based on this protocol, the Guangdong Earthquake Monitoring Center developed the JEEW and integrated it into JOPENS6 [27], while the Fujian Earthquake Administration developed the FJEEW [28], forming a multi-stakeholder technical landscape.

During the Fourteenth Five-Year Plan (2021–2025), construction of the national EEW network has accelerated, and EEWSs have been deployed across 31 provinces [29]. CSTP has undergone multiple rounds of revision and refinement and has been incorporated as a standard feature in intensity meters produced by major Chinese manufacturers [18,30]. It has also begun to be supported by some broadband and short-period seismometers. If CSTP ultimately becomes the standard configuration, existing processing systems will need to support only this single protocol to efficiently receive real-time data from new instruments, fundamentally resolving protocol-compatibility issues.

Currently, the only publicly available CSTP server is the ComServ2Server module in JOPENS6 [26]. Although JOPENS6 is feature-rich, mature, and stable, it runs only on the FreeBSD operating system, requires complex installation and configuration, and uses module-based licensing, resulting in high deployment and operational costs. In addition, JOPENS6 exposes data services solely via NetSeis/HTTP protocol, the HTTP variant of NetSeis/IP. For temporary network scenarios—such as earthquake emergency response or seismic arrays—where the main goals are to verify instrument operation, archive real-time data, or feed data into existing processing systems (e.g., JOPENS5, SeisComP, Earthworm, Antelope), there is still a lack of a simple, low-cost solution for integrating CSTP-enabled instruments.

**Figure 2:**
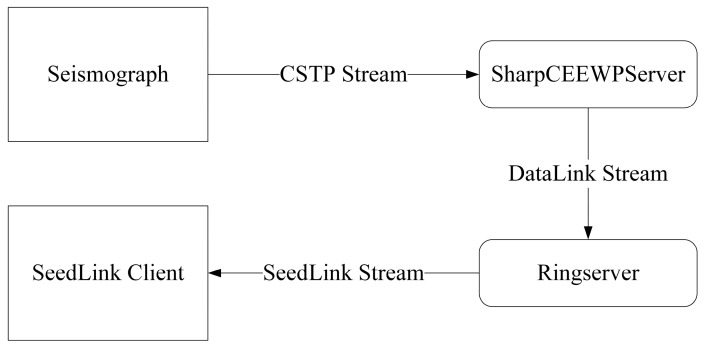
Concept of the two-stage data forwarding system composed of the proposed lightweight server SharpCEEWPServer and the RingServer. Rectangles represent the data sources or destinations, rounded rectangles represent the data transformation processes, and arrows indicate the data flow.

Therefore, it is of practical value to develop a lightweight, graphical server that can be flexibly deployed and used out-of-the-box. Such a server should not only provide basic functions such as client connection management, remote control, real-time data reception, visualization, and archiving, but should also be able to convert CSTP real-time data streams into widely supported SeedLink streams to enable seamless integration with existing processing systems. To avoid the additional workload and technical risk of independently implementing a SeedLink server, this study adopts the mature and stable RingServer as the external data-service interface [31]. RingServer supports the unidirectional SeedLink protocol as well as the bidirectional DataLink protocol. Consequently, as illustrated in Figure 2, the target server only needs to implement a DataLink client and can then rely on RingServer to deliver SeedLink real-time data streams, substantially reducing development effort and cost. Based on these requirements, this study designs and implements a CSTP-based server, SharpCEEWPServer, providing a simple and low-cost solution for integrating new instruments into temporary seismic networks.

## 2. CSTP Overview

CSTP is an application-layer protocol based on TCP/IP. Because its specification has not yet been formally published and to highlight its core characteristics, this paper discusses only the application layer, focusing on its design concepts and differences from other protocols. The core elements of a seismic communication protocol can be broadly divided into three modules:

- Session flow: Defines the complete sequence of events after a client and server establish a connection, including handshake, control command transmission, data transmission, and termination;
- Command format: Specifies control instructions during the session, excluding data, such as mode switching and parameter configuration.
- Data format: Defines how waveform and non-waveform data are encapsulated.

From a data-flow perspective, CSTP is a client-to-server unidirectional protocol for transmitting observational data, which differs from most existing protocols. Its main advantage is reduced reliance on static IP address resources. Even when a seismometer with an embedded CSTP client is deployed on a public mobile network behind multiple layers of Network Address Translation (NAT), it can still establish a connection to the server, improving the flexibility and environmental adaptability of network deployments.

### 2.1. Session

**Figure 3:**
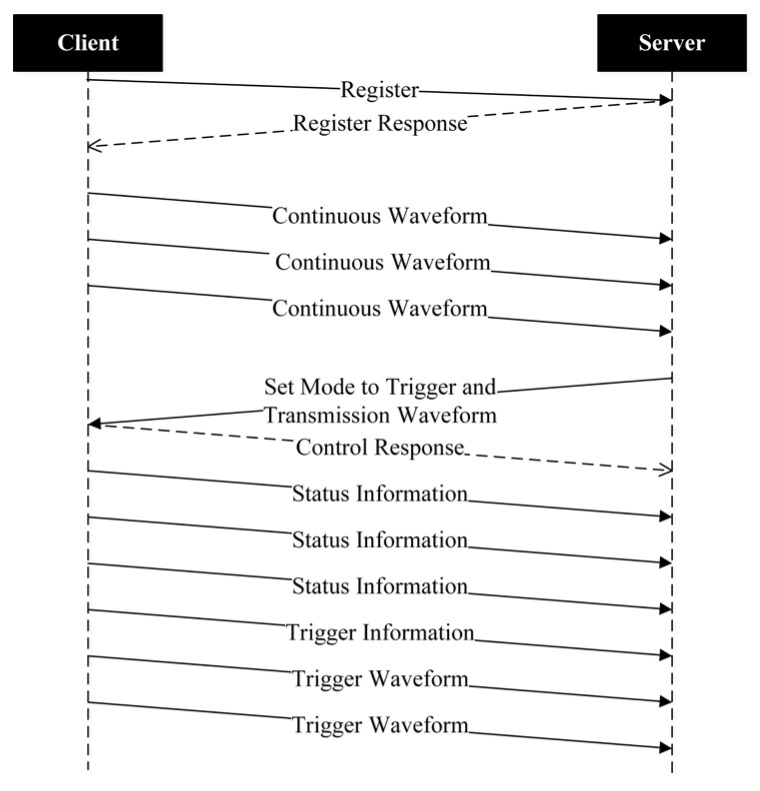
Sequence diagram of a typical CSTP session. The client is on the left, and the server is on the right. The dashed lines represent their lifelines, while solid lines represent sent messages, and dashed lines represent responses to those messages.

Figure 3 illustrates a typical CSTP session. After a TCP connection is established between the client and the server, the client immediately sends a registration request. Once the server has authenticated the client and returned a response, the client starts sending data without waiting for an explicit command, unlike many other protocols. During the session, the server can send control commands to the client, and the client must return a response containing the execution result, regardless of whether the operation succeeds or fails. To reduce transmission latency and improve communication efficiency, the server does not acknowledge observational or status data packets sent by the client. The session is terminated when either side closes the TCP connection; no explicit termination command is required.

### 2.2. Command

Earlier protocols such as SeedLink3 and NetSeis/IP largely followed the command model of LISS, sending plaintext commands over Telnet sessions, which poses serious security risks. Later protocols abandoned Telnet entirely: Earthworm and SeedLink 4 use text-based commands, and DataLink uses a combination of text and binary commands. CSTP, similar to CD-1, uses purely binary commands. All of these protocols can be operated over encrypted channels to improve security.

To reduce latency and bandwidth usage, CSTP employs a compact packet structure with lengths constrained to powers of two. In early versions, command packets had a fixed length of 64 bytes, and data packets had a fixed length of 256 bytes and carried less than one second of waveform data to reduce latency; real-time streams using such packets are referred to as “fast streams” [27]. In contrast, other data packets with lengths of 512 bytes or more can contain several seconds of waveform data and therefore have larger latency; real-time streams using these packets are referred to as “slow streams”.

### 2.3. Data Format

Seismic data communication protocols can be categorized into two groups according to the waveform data packet format:

- protocols that transmit custom data formats, such as CD-1 and Earthworm;
- protocols that are based on the SEED format and transmit MiniSEED packets, including LISS, SeedLink, NetSeis/IP, and DataLink [32].

CSTP also belongs to the second group, but differs from other protocols in that it neither forwards raw MiniSEED packets directly nor simply adds an external protocol header. The first 6 bytes of the MiniSEED packet, originally encoded in ASCII to record the packet sequence number, have the first 2 bytes replaced with a packet type identifier, while the remaining 4 bytes’ 32 bits are split into 3 bits for indexing the packet length and 29 bits representing the packet sequence number as an unsigned integer. Additionally, CSTP’s waveform data packets include a Blockette 1002, which is not present in the SEED standard, to record dimensional units and sensitivity coefficients. MiniSEED has good self-explanatory properties, as its Blockette 1000 contains essential decoding information such as data encoding format, byte count, and packet length. Therefore, by removing CSTP-related information, replacing the first 6 bytes of the packet with the ASCII-encoded packet sequence number, and deleting Blockette 1002, a standard MiniSEED packet can be obtained for archiving or for forwarding after adding the DataLink header.

## 3. Design of Architecture

**Figure 4:**
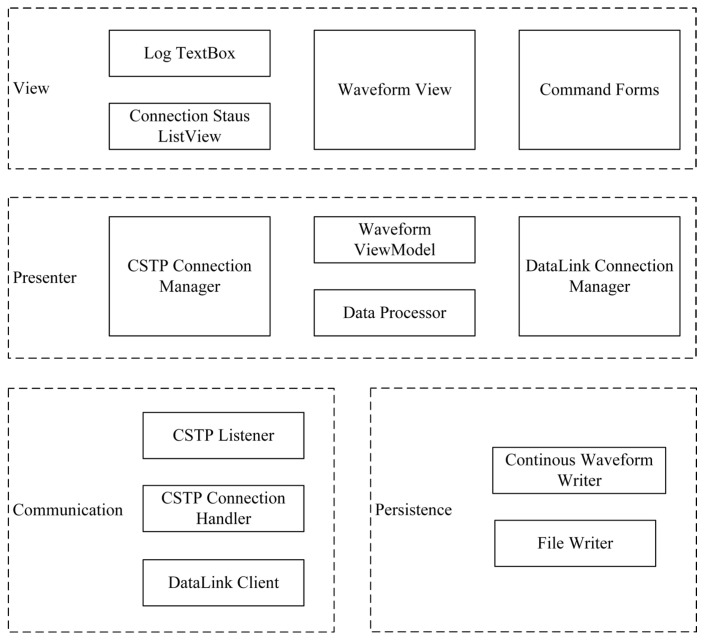
Overall architecture of the SharpCEEWPServer.

The lightweight server designed in this study is a monolithic application built on the .NET 8 development environment with a WinForm-based frontend. It adopts a three-layer architecture consisting, from bottom to top, of the Communication and Persistence Layer, the Presenter Layer, and the View Layer. The overall architecture is shown in Figure 4.

### 3.1. Communication and Persistence Layer

The communication layer establishes network connections with external devices and handles data transmission. Apart from basic operations such as packet concatenation, segmentation, and classification, it performs no complex processing. Instead, packets are passed directly to the Presenter Layer to avoid blocking the socket.

The CSTP Listener listens for incoming connections from clients such as seismometers, software applications, or other data servers. Once a registration packet is received, the connection object and the registration information are handed off to the CSTP Connection Manager in the Presenter Layer, which performs authentication and generates the corresponding response packet. After successful registration, command and data exchange with the client is handled by the CSTP Connection Handler. Control commands sent to the client are generated by the CSTP Connection Manager, whereas responses and data received from the client are forwarded to the Data Processor; the CSTP Connection Handler itself does not perform any substantive processing. The DataLink Client is responsible for connecting to the RingServer and sending real-time data streams, and therefore implements only the essential DataLink commands, such as ID and WRITE.

The persistence layer receives various data packets from the Data Processor and writes them to the appropriate files on disk. Continuous waveform data are the most important and highest-frequency data in seismic monitoring. To avoid I/O resource contention, all continuous waveform data are routed to a dedicated Continuous Waveform Writer for centralized management and writing. Low-frequency data, such as status packets, trigger information, and event waveforms, are managed and written through a File Writer object owned by the Data Processor.

### 3.2. Presenter Layer

**Figure 5:**
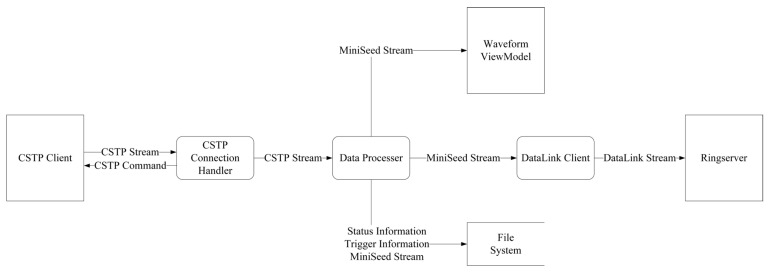
Data processing pipeline of real-time data flow inside SharpCEEWPServer. Rectangles represent data sources or destinations, rounded rectangles represent data transformation processes, open rectangles represent data storage, and arrows indicate the data flow.

The Presenter Layer implements the core business logic of the software and acts as an intermediary between the communication, persistence, and view layers, preventing direct interaction and data transfer between these layers. The CSTP Connection Manager manages all client connections, handling registration validation, status monitoring, reconnection management, and control command generation. Similarly, the DataLink Connection Manager manages all DataLink clients. As show in Figure 5, the Data Processor is the core data-processing module of the server: it receives data packets from the CSTP Connection Handler, classifies and converts them into appropriate formats, and forwards them to other modules—for example, to the Continuous Waveform Writer for disk storage, to the Waveform ViewModel to update the view-layer cache, or to the DataLink Client for encapsulation and transmission to the RingServer. The Waveform ViewModel is not a ViewModel in the traditional MVVM sense; instead, it manages the cache used by the real-time waveform display controls. This design is motivated by the fact that WinForm and other graphical frameworks render the user interface exclusively on the main thread. Offloading cache management and related non-rendering tasks from the main thread is essential for improving graphical performance.

### 3.3. View Layer

The view layer renders data and client status into graphical user interfaces and provides interactive operations for users. Log messages are displayed in a Log TextBox and, in more detailed form, written simultaneously to log files to facilitate troubleshooting. The Connection Status ListView presents the status of all CSTP clients managed by the CSTP Connection Manager in tabular form. Command Forms are a set of simple windows used to configure command parameters; these parameters are passed to the CSTP Connection Manager to construct command packets, which are then sent to CSTP clients via the CSTP Connection Handler.

The Waveform View is the most complex component of the view layer. It displays incoming real-time waveform data in a cyclic, overwriting manner (rolling window). Because waveform packets may arrive out of order, contain gaps, partially overlap, or be duplicated, a custom Waveform View module is required. Rendering is separated from non-rendering logic, with the latter delegated to the Waveform ViewModel in the Presenter Layer to improve performance.

Through this layered architecture, network communication, data storage, business logic, and graphical display are clearly separated into different tiers, and each module has well-defined responsibilities and loose coupling. The communication and persistence layers handle only external data exchange and are unaware of the Presenter and View Layers. The Presenter Layer focuses on business logic and maintaining the data cache required by the View Layer, without concern for data sources or the specific graphics library used. The View Layer updates the graphical interface using timer events, without directly accessing the communication or persistence layers and without intruding into the Presenter Layer. This design not only facilitates replacement of individual module implementations, but also isolates potential bottlenecks—such as network I/O, data processing, file writing, graphics buffer updates, and rendering—into separate components. Combined with asynchronous and multithreaded processing, this helps ensure robust performance under high-concurrency workloads.

Through a well-structured architecture, this software design divides network communication, data storage, business logic, and graphical display into distinct layers. Each module has a clear function and is decoupled from the others. The communication and persistence layers are responsible solely for external data interactions and are unaware of the Presenter Layer and the view layer. The Presenter Layer handles business logic and the data cache needed to update the view layer, without concern for data sources or which graphical library is used. The view layer updates the graphical interface through timer events, without directly interacting with the communication or persistence layers, and does not interfere with the Presenter Layer. This architectural design not only facilitates the replacement of specific implementations but also isolates potential bottlenecks—such as network communication, data processing, file writing, graphics buffer updates, and graphical rendering—into separate modules, handled by individual threads. Combined with asynchronous processing and thread pool techniques, this design ensures the system can efficiently manage high-concurrency scenarios.

## 4. Implementation

We implemented SharpCEEWPServer using the .NET 8 development environment, the WinForms graphical framework, and the ScottPlot plotting library. The communication, persistence, and presenter layers together provide all server-side functionality except the graphical interface and are implemented solely with native .NET 8 libraries. As a result, this core can be refactored into a pure console application, used with alternative view-layer implementations, or compiled as a dynamic-link library and embedded into other .NET 8 applications. The view layer is implemented with WinForms and ScottPlot and updates the graphical interface using timer-driven refreshes to reduce load on the main UI thread. WinForms was chosen mainly to accelerate development; future versions are planned to use a cross-platform graphical framework to support Linux-based deployments.

Each module in the software runs in its own thread. Modules exchange data through first-in-first-out (FIFO) queues, while asynchronous operations are used for internal read, write, and processing tasks. Thread allocation and task scheduling are managed automatically by the .NET thread pool. Runtime parameters are configured via a JSON file, which is easy to edit and back up. In typical use, only a small number of fields need to be adjusted, such as CSTP client credentials, the archive root directory, the RingServer IP address, and the DataLink service port number.

**Figure 6:**
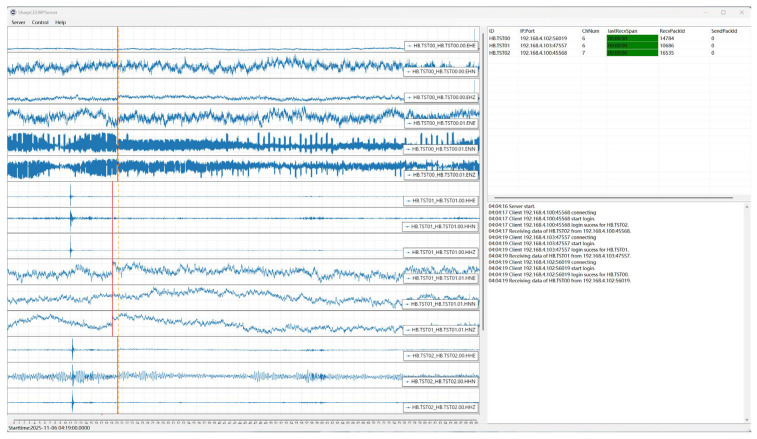
MainForm of SharpCEEWPServer. The **left panel** is the waveform display area, the **upper-right** is the client connection status list, and the **lower-right** is the log display area. The red vertical dashed line in the waveform display area represents the waveform update time, and the red vertical solid line represents the data end time.

The main form of SharpCEEWPServer is shown in Figure 6. The left panel is the waveform display area corresponding to the Waveform View module in the view layer, where each trace represents a single seismic data channel. Because waveform packets may arrive out of order, contain gaps, partially overlap, or be duplicated, no existing off-the-shelf control could fully meet our requirements. We therefore implemented a custom waveform control on top of ScottPlot. To support high-concurrency scenarios, we apply a visualization virtualization technique to reuse a limited number of controls when displaying many channels. For bursty connection and registration events, channel information is updated using delayed, batched operations to avoid frequent, expensive refreshes. Finally, timer-based updates are used to reduce the redraw frequency of individual controls. These targeted optimizations significantly improve GUI performance under high-load conditions.

**Figure 7:**
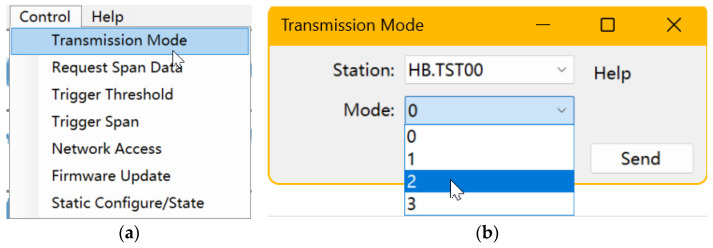
Command-sending interface: (**a**) Commands menu; (**b**) Command Parameter Configuration Form.

The upper-right area of the main window contains the connection status list, implemented as a simple ListView control (Connection Status ListView) that periodically refreshes the status of all clients. The lower-right area is the log display (Log TextBox), which shows log messages produced by the Serilog library in real time. As illustrated in Figure 7, the Control menu in the main window provides seven command options that can be sent to connected clients; responses returned by the clients are printed in the log display.

During the development and improvement of the server, three main versions were created: Version 1 was a prototype program to validate feasibility, without a layered architecture. Each thread handled a single client connection, and the tasks of receiving, processing, and storing packets were executed sequentially. The waveform display rendering and background cache updates all ran on the main thread. Version 2 improved upon this by adopting the architecture described in this paper but did not optimize the performance of the waveform display controls. Version 3, which is described in this paper, utilized batch updates, delayed processing, and virtualization techniques to optimize graphical performance. The performance differences between the three versions were significant. In Version 1, the graphical interface failed to work properly when processing real-time data streams from more than 50 stations. Version 2, when handling about 600 stations, could not complete the initialization of the waveform display components.

**Figure 8:**
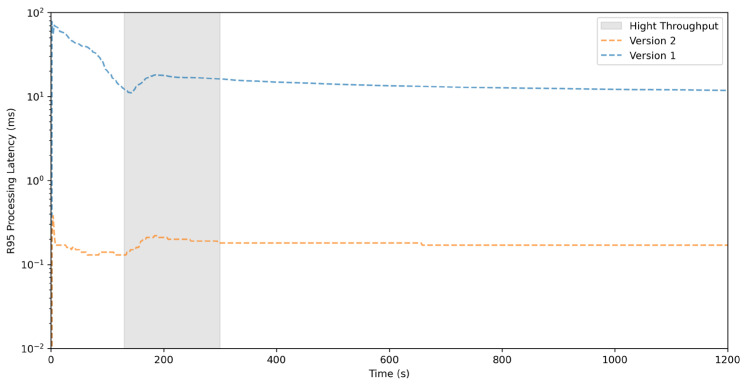
R95 latency of Version 1 without the layered architecture and Version 2 with the layered architecture when processing real-time data streams from 30 three-component seismometers.

Therefore, using the same method as described in Section 5.3, the performance of Versions 1 and 2 was tested using the 30 stations closest to the epicenter. As shown in Figure 8, Version 1, which did not use the layered architecture, the R95 (95th percentile) processing latency from when the server receives the packet to before it is written to disk peaks at 78 ms during control initialization, which dropped to 11 ms after about 140 s, but when the throughput peak reached only 73 KB/s, the latency increased again to 18 ms. In contrast, Version 2, which was re-implemented with the layered architecture described in this paper, showed R95 processing latency as indicated by the orange dashed line, with the waveform control initialization phase taking only 0.53 ms, and the throughput peak reached a latency of just 0.22 ms, a nearly 100-fold improvement. This demonstrates that by using the architecture described in this paper, separating blocking points such as TCP connection handling, data decompression, waveform display control rendering, and control cache updates into independent threads for asynchronous execution can significantly improve program performance, even without other optimizations.

In summary, the implemented server software is portable, easy to configure, and provides an intuitive graphical interface. Rather than relaxing performance requirements because typical temporary networks often include only a few dozen instruments, we specifically optimized both the architecture and the implementation for high-concurrency scenarios.

## 5. Tests

### 5.1. Hardware Compatibility Tests

Since its initial proposal, the CSTP protocol has undergone multiple revisions, and different models from different manufacturers may support different protocol versions and feature sets. To evaluate the hardware compatibility of the server implemented in this study under realistic conditions, we selected several commercial integrated seismometers and seismic digitizers as test devices and examined their interoperability with the server in terms of registration, data transmission, and remote control. In this subsection, compatibility for a given feature is defined as follows: when the server sends a control request to a device, or when the device sends data to the server, the counterpart can correctly execute the corresponding action or correctly parse and process the received data.

**Figure 9:**
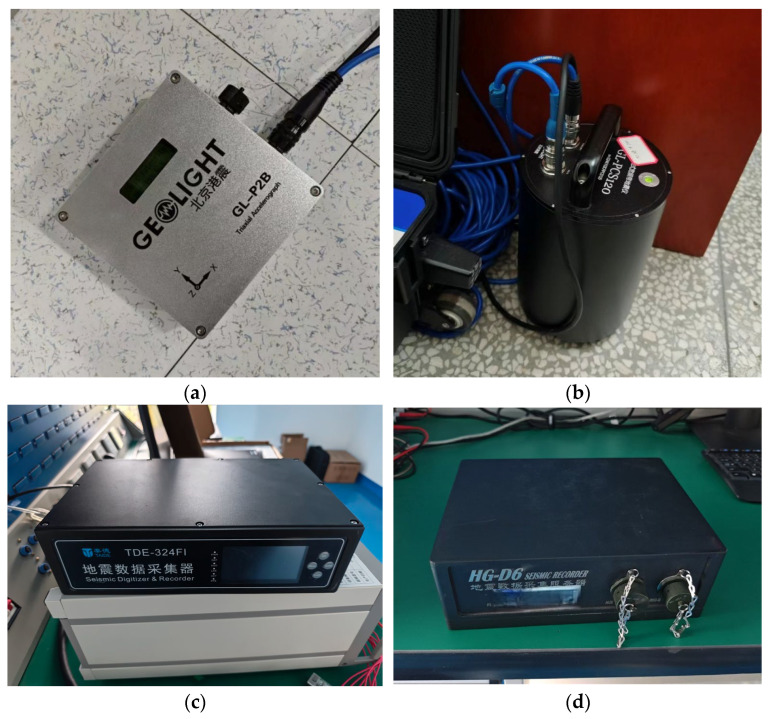
Instruments used in the compatibility tests: (**a**) GL-P2B integrated triaxial accelerograph (北京港震, Geolight); (**b**) GL-PCS120 portable broadband seismograph; (**c**) TDE-324FI seismic digitizer (地震数据采集器, Seismic Digitizer & Recorder); (**d**) HG-D6 seismic digitizer (地震数据采集服务器, Seismic Recorder). The Chinese terms are directly paired with their English translations in the image.

The tests used four models currently available on the Chinese market: the TDE-324FI seismic digitizer (Zhuhai Taide Enterprise Co., Ltd., Zhuhai, Guangdong, China), the GL-P2B integrated accelerometer andthe GL-PCS120 portable broadband seismometer (Beijing Gangzhen Technology Co., Ltd., Beijing, China), and the HG-D6 seismic digitizer (Zhongzhen Huachuang Technology Co., Ltd., Shenzhen, Guangdong, China). The TDE-324FI, GL-P2B, and HG-D6 are widely used accelerometers or associated seismic digitizers, each from one of the three main seismometer manufacturers in China. The GL-PCS120 is a portable broadband seismometer primarily used for seismic array observations. Except for the relatively older HG-D6, the TDE-324FI, GL-P2B, and GL-PCS120 were all newly purchased within the past two years. These four devices, representing different manufacturers, production years, and observational bandwidths, are typical of the equipment used for deploying temporary networks during seismic observations, making them highly representative. The appearance of the device is shown in Figure 9.

As summarized in Table 1, all four devices were able to complete the CSTP registration procedure with the server and to send real-time data streams in continuous waveform mode. Using each manufacturer’s configuration software, we attempted to switch the devices into the three transmission modes defined in the CSTP specification: continuous waveform mode, triggered mode with waveform transmission, and triggered mode without waveform transmission. TDE-324FI supports all three modes and sends data packets consistent with each mode. GL-P2B and GL-PCS120 support continuous mode and triggered mode with waveform transmission, but not triggered mode without waveform transmission. For HG-D6, we attempted to switch the transmission mode via the vendor software but were not successful.

Differences among devices are even more pronounced for remote control. TDE-324FI correctly executes most control commands but does not return the response packets required by the specification. GL-P2B and GL-PCS120 neither execute the commands nor return any response. HG-D6 disconnects from the server and immediately reconnects whenever it receives a control request.

Overall, these results indicate that all tested devices can establish a stable CSTP session with SharpCEEWPServer, complete registration, and send continuous real-time waveform data, which already satisfies the core functional requirements for temporary networks and data forwarding applications. By contrast, support for extended features such as transmission-mode switching and remote control varies considerably across models. This inconsistency is more likely due to differences in CSTP protocol versions, early models implementing only a subset of features, or divergent interpretations of protocol details by manufacturers, rather than issues in the server implementation itself. It also suggests that CSTP implementations are not yet fully uniform, and achieving comprehensive interoperability may require the standard to be finalized and officially released.

### 5.2. Real-Time Data Stream Forwarding Tests

**Figure 10:**
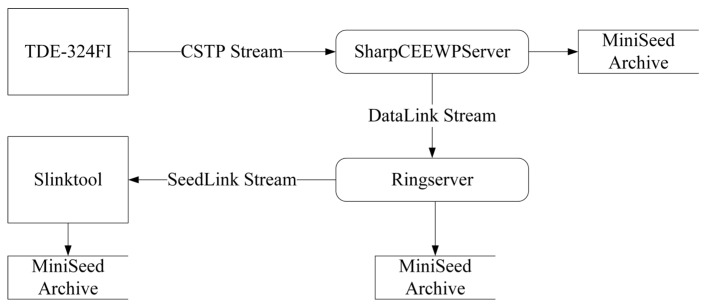
Architecture of the real-time data stream forwarding system. Rectangles represent data sources or destinations, rounded rectangles represent data transformation processes, open rectangles represent data storage, and arrows indicate the data flow.

To evaluate the correctness and stability of the server’s real-time data forwarding functionality, we built a forwarding chain that emulates a production environment, as shown in Figure 10. Each node in the chain runs on a different hardware platform and they are interconnected via the internet. The data source is a TDE-324FI seismic digitizer configured to send six real-time channels, with each channel producing one 512-byte waveform packet per second containing 100 samples. SharpCEEWPServer is deployed on a laptop in the same local area network as the TDE-324FI. It receives the incoming CSTP real-time data stream and forwards it, after conversion, to RingServer via a DataLink client. RingServer is deployed on a cloud server with a public IP address; it receives the DataLink real-time data stream and exposes it to external clients via its SeedLink server. slinktool—a commonly used SeedLink testing utility—is deployed on a Precision T7920 workstation and acts as a SeedLink client to retrieve the SeedLink data stream from RingServer [33]. SharpCEEWPServer, RingServer, and slinktool all enable data archiving, and consistency of the archived MiniSEED packets across the three nodes is used to verify correct end-to-end operation.

To avoid packet loss caused by downstream nodes not running, we start the components in the following order at the beginning of the test: SharpCEEWPServer, RingServer, and slinktool, and finally the TDE-324FI. At the end of the test, we first stop the TDE-324FI, then shut down SharpCEEWPServer, slinktool, and RingServer in sequence after all packets have been received. In total, SharpCEEWPServer archives 320,112 MiniSEED packets spanning UTC 2025-08-12T09:10:48.00 to 2025-08-12T23:59:59.99, with sequence numbers from 1 to 320,112. RingServer and slinktool archive exactly the same set of MiniSEED packets. For a single channel, the elapsed time between the first and last sample is 53,352 s; with six channels and one packet per channel per second, the theoretical total number of packets is 320,112, consistent with what is archived at each node. Using ObsPy to parse the archived data from each node, no parsing errors were encountered, and complete continuous waveforms were obtained, confirming the integrity and correctness of the data.

These results demonstrate that the forwarding system in Figure 10 can reliably convert CSTP real-time data streams into MiniSEED files and continuously transform them into standard SeedLink real-time data streams for downstream consumption. In other words, existing processing systems such as JOPENS5, SeisComP, and Earthworm can interoperate with new CSTP-capable instruments without modification by using this forwarding scheme.

### 5.3. Concurrency Performance Tests

Because only a limited number of CSTP-compatible broadband seismometers were available in our group, we developed a dedicated simulator, SharpCEEWPSender, to emulate a temporary network for concurrency testing. SharpCEEWPSender reads archived waveform files from disk, creates one CSTP client per station, registers each client with the server, and then sends packets at 1 s intervals. It can also generate virtual stations by duplicating existing station data. To avoid external interference, all concurrency tests were conducted in a local-area network (LAN) with only two transmission nodes: SharpCEEWPSender, which simulated hundreds to thousands of stations, and SharpCEEWPServer, which received and archived continuous waveform data without forwarding.

SharpCEEWPSender was deployed on a DELL Precision T7920 workstation equipped with an Intel Xeon Gold 6248R CPU (Intel, Santa Clara, CA, USA), 256 GB RAM(Samsung, Seoul, Republic of Korea), and a 2 TB SSD, using the onboard Intel I219 gigabit Ethernet adapter (Intel, Santa Clara, CA, USA). SharpCEEWPServer was deployed on an Asus Zenbook UX8402V laptop (Asus, Taipei, Taiwan) with an Intel Core i9-13900H CPU (Intel, Santa Clara, CA, USA), 32 GB RAM(Samsung, Seoul, Republic of Korea), a 1 TB SSD, and a 2880 × 1800 display at 120 Hz, running Windows 11 Home Edition and using an external ASIX AX88179 gigabit Ethernet adapter (ASIX, New Taipei City, Taiwan). Both computers were connected via wired Ethernet to a HUAWEI AX3 Pro (Huawei, Shenzhen, China) router, forming a 1 Gbit/s LAN.

**Figure 11:**
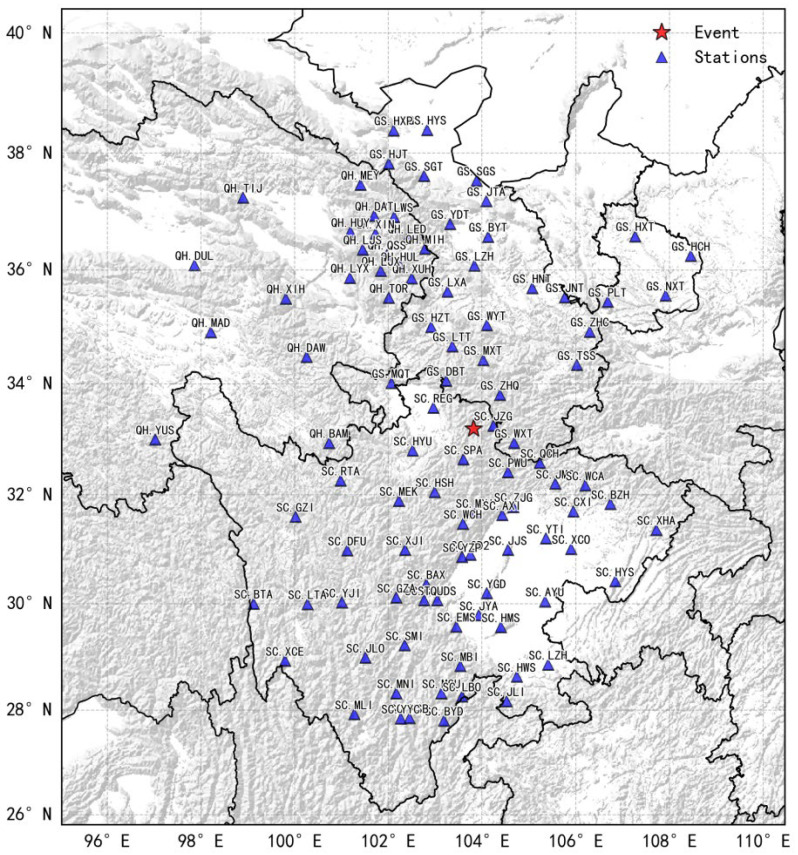
Epicenter and station distribution used in the concurrency tests. The red pentagram represents the epicenter location of the Jiuzhaigou M7.0 earthquake, and the blue triangles represent the locations of the real stations used in simulating real-time waveforms for the test.

Waveform data from seismometers are typically compressed using Steim algorithms, with the compression ratio varying dynamically with the differences between adjacent samples. Smaller differences yield higher compression ratios, whereas strong ground motion leads to lower compression ratios [32,34]. To approximate a realistic high-load condition, we used real data from a major earthquake. We selected 100 stations located closest to the epicenter of the 2017 Jiuzhaigou M7.0 earthquake from regional networks in Sichuan, Gansu, and Qinghai. From the China Earthquake Networks Center (CENC) archives, we extracted 1200 s of continuous waveform data, from 100 s before to 1100 s after the origin time. The epicenter and station distribution (maximum epicentral distance of approximately 640 km) are shown in Figure 11.

**Figure 12:**
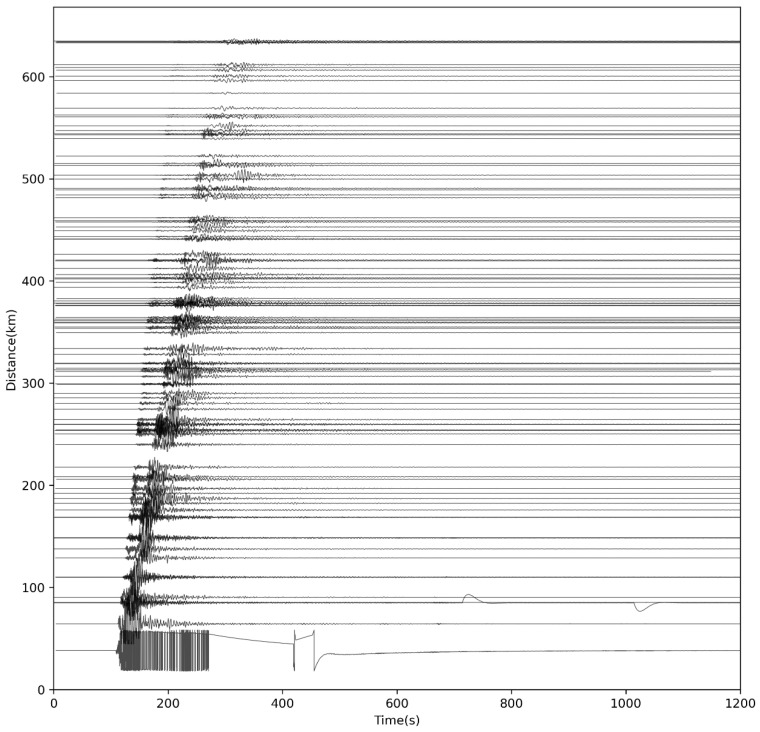
Waveforms of the 2017 Jiuzhaigou M7.0 earthquake used in the concurrency tests. The solid line represents the vertical component of the waveform recorded by each station in the network, normalized according to the maximum amplitude of all stations and magnified by a factor of 20.

The archived waveforms used in the tests are shown in Figure 12. Based on these 100 stations and the station-duplication function in SharpCEEWPSender, we simulated 200–2000 stations within 640 km of the epicenter, all registering with SharpCEEWPServer and sending real-time waveform data corresponding to the M7.0 event. Taking the example of simulating 200 stations and the SC.JZG seismic station, which is the closest to the epicenter, SharpCEEWPSender first scans and sorts the archived data of SC.JZG. It then calculates the time difference between the current time and the reference time—the time 100 s before the origin of the Jiuzhaigou earthquake—as the reference time interval. Two new clients are created and assigned the IDs SC.JZG00 and SC.JZG01, and each is placed in a separate thread for execution. After the clients complete the registration, events are triggered at 1-s intervals, sequentially extracting the sorted SC.JZG archived data packets. If the time of the data packet plus the reference time interval is less than the current time, the packet is immediately sent; otherwise, it waits until the next second for evaluation. Before sending, the data packet is modified to match the client’s network and station names, the start time is adjusted by adding the reference time interval, and it is then converted into a CSTP packet. The same process applies to other stations and additional virtual seismic stations. During each test, throughput, CPU usage, and memory usage were recorded at 2 s intervals. In addition, the mean processing latency and 95th-percentile (R95) processing latency—measured from packet reception to just before the packet was written to disk—were computed to evaluate concurrency performance.

**Figure 13:**
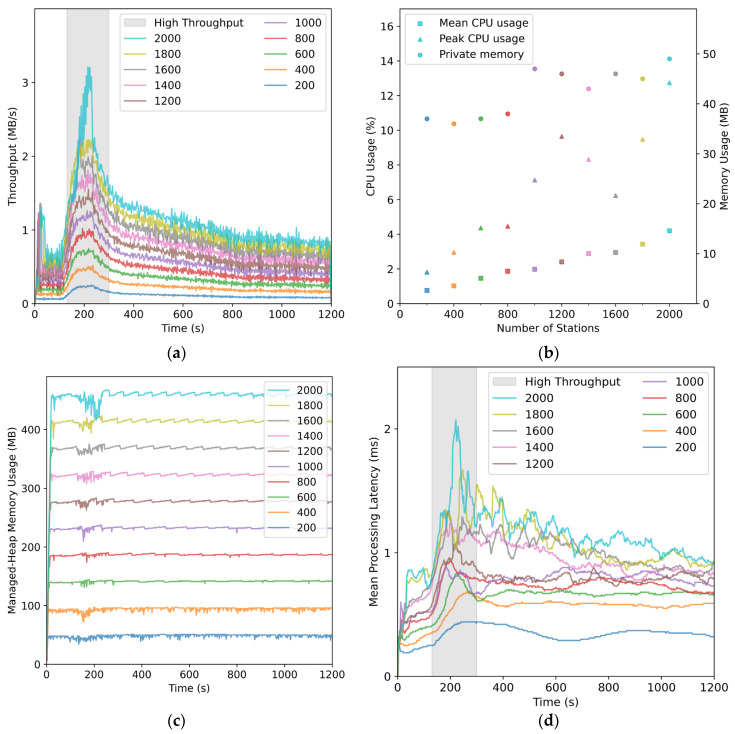
Concurrency performance metrics of SharpCEEWPServer under increasing station counts: (**a**) throughput over time; (**b**) CPU usage (mean and peak) and private memory usage; (**c**) managed-heap memory usage; (**d**) mean processing latency.

As shown in Figure 13a, SharpCEEWPServer experienced a brief throughput peak immediately after SharpCEEWPSender initiated connections, due to a large number of registration packets and accumulated data. It then entered a steady state, receiving only background noise. When seismic phases began arriving at the simulated stations (130–300 s), throughput increased again, forming a higher and longer-lasting peak before gradually returning to background levels. With 200 stations, the quiet-period throughput was about 65 KB/s, corresponding to an overall compression ratio of approximately 3.6:1, indicating that compressed packets were less than one third the size of the uncompressed data even after including header information. During the 130–300 s peak interval, the maximum throughput reached about 240 KB/s and the compression ratio dropped to approximately 0.97:1, so that the compressed packets were slightly larger than the raw data.

Figure 13b shows CPU usage sampled during the 130–300 s interval. As the station count increased, the mean CPU usage rose smoothly from 0.75% to 4.19%, while the peak CPU usage increased from 1.81% to 12.74%. The private memory usage remained around 40 MB for up to 800 stations and increased to approximately 50 MB when the number of stations exceeded 1000. Figure 13c shows the managed-heap memory usage, which increased by about 46 MB for every additional 200 stations, reaching roughly 460 MB at 2000 stations, with only minor fluctuations during each test. Figure 13d shows that the mean processing latency increased gradually with station count, from a maximum of 0.88 ms at 1000 stations to 2.07 ms at 2000 stations.

**Figure 14:**
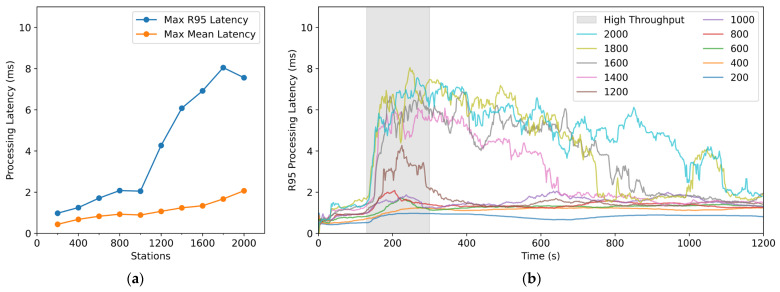
R95 (95th percentile) processing latency for different station counts: (**a**) Maximum R95 and maximum mean processing latency for different station counts; (**b**) Time evolution of R95 processing latency for different station counts.

Although the mean latency remained low, Figure 14a,b show that the R95 processing latency exhibited nonlinear growth and increasing variability as the number of stations increased. For up to 1000 stations, R95 latency closely followed the throughput evolution and returned to baseline quickly after the load decreased. At 1200 stations, the R95 peak increased from about 2 ms to about 4 ms, but still dropped rapidly when the load declined. At 1400 stations, the R95 peak further increased to about 6 ms and began to show a pronounced long-tail effect. At 2000 stations, the sharp increase and long-tail behavior persisted until near the end of the test.

**Figure 15:**
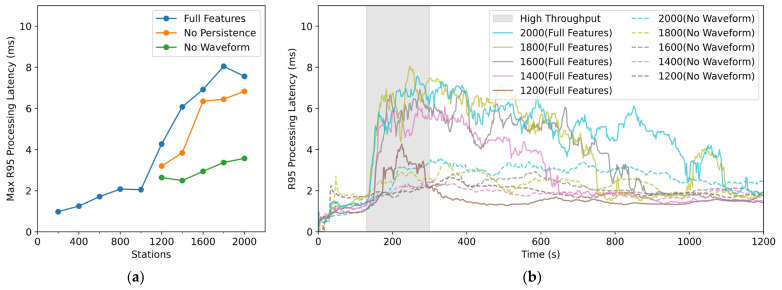
Comparison of R95 processing latency: (**a**) Maximum R95 processing latency for different features; (**b**) Time evolution of R95 processing latency for different features.

Overall, even in the extreme scenario of receiving real-time data from 2000 three-component stations, SharpCEEWPServer kept CPU and memory usage well below the commonly adopted 70% safety threshold, and maintained the mean processing latency below 2 ms, indicating high processing efficiency. However, the sharp increase in R95 latency above approximately 1200 stations and the pronounced long-tail effect beyond 1400 stations suggest that the server approaches saturation under the load induced by an M7.0 earthquake. The sharp increase in R95 latency, along with low CPU and memory usage, suggests that the performance bottleneck may arise from other factors such as GC overhead, disk I/O, UI rendering load, or thread scheduling, rather than computational resources. The potential major bottlenecks could be twofold: the continuous waveform data archiving module (Continuous Waveform Writer) and the waveform rendering module (Waveform View) along with its cache management module (Waveform ViewModel). Therefore, by disabling continuous waveform disk writing and waveform rendering with background cache management, the metrics for 1200 to 2000 stations were retested, and the results are shown in Figure 15. As seen in Figure 15a, the maximum R95 processing latency from 1200 to 2000 stations shows only a slight decrease when disk writing is disabled, but a significant drop when waveform rendering is disabled, smoothing the data to levels comparable to those seen with fewer than 1000 stations. The dashed line in Figure 15a represents the R95 processing latency curve when waveform rendering is disabled, showing a slight increase during throughput peak intervals, with the sharp increase and long-tail effects disappearing. Therefore, it can be concluded that the main cause of the sharp R95 latency increase and long-tail phenomenon is real-time waveform rendering and its background cache management.

We also tested the scenario where the waveform rendering module, Waveform View, was disabled while keeping the cache management module, Waveform ViewModel, running. In this case, the R95 latency only decreased slightly. The main cause of this phenomenon may be that the Waveform ViewModel generates a large number of temporary objects when decompressing Steim-encoded compressed data, which increases the frequency of garbage collection (GC), as well as lock contention when updating the cache and rendering the waveforms. Therefore, in actual production environments, disabling waveform rendering and background cache management can improve performance.

In summary, when the number of stations reaches 1200, the peak R95 processing latency can decrease rapidly with throughput, and even when the number of stations reaches 2000, the server is still able to recover after extended operation. For the sake of maintaining a certain performance margin, when using laptop-class hardware to archive real-time data streams, even under ideal conditions, the number of real-time data streams from three-component seismometers that the server can handle should be kept below 1000. Of course, the target of SharpCEEWPServer is to address temporary network scenarios with a scale ranging from a few to several dozen seismometers. For permanent networks or large scientific arrays with more than 100 seismometers, or earthquake early warning systems, it is still recommended to use distributed commercial data processing systems.

### 5.4. Network Impairment Testing

4G mobile networks are commonly used for real-time data transmission in temporary seismic networks and earthquake early warning systems, but their stability is far inferior to that of wired networks. In this section, the network diagnostic tool clumsy is used within a local area network to simulate a poor mobile network by introducing 100 ms of latency, 5% packet loss, and 10% out-of-order packets. A 100% packet loss scenario is also simulated to represent temporary disconnections, and the results are compared with data from tests conducted under normal network conditions. SharpCEEWPSender is deployed on a DELL Precision T7920 workstation, using archived data from the LTT station, which has a relatively even packet distribution, to simulate real-time data streams. The packet start times are modified to match the transmission time before sending. SharpCEEWPServer is deployed on an Asus Zenbook laptop, receives, displays, and archives the real-time data stream, while recording the timing information during processing.

In all tests, SharpCEEWPServer ran stably and quickly recovered from disconnections. The test results are shown in Table 2. In normal mode, all packets were transmitted correctly, with an R95 transmission latency of 110.76 ms, an R95 processing latency of 0.29 ms, and an R95 end-to-end latency from sending to completion of 110.98 ms. When both software programs run on the same computer and communicate via the local area network IP, the transmission latency is below 1 ms. Therefore, the transmission latency of 110.76 ms in the benchmark test is caused by clock desynchronization. In the simulated unstable mobile network, all data were transmitted completely, but the R95 transmission latency significantly increased to 651.91 ms, and the processing latency rose to 0.52 ms. During 10 temporary disconnections lasting over 3 s, an average of about 4 packets were lost at the application layer, and both transmission latency and processing latency showed slight increases.

Based on the above test results, it can be seen that SharpCEEWPServer can operate stably in unstable network environments and quickly recover from network anomalies. While prioritizing the clearing of problematic buffers ensures the software’s stability and allows it to quickly recover from network anomalies, this simple and conservative strategy also causes some data packets to be lost, compromising the integrity of archived data. Therefore, optimizing the network transmission latency for low-frequency data and implementing the retransmission of lost packets based on the CSTP data request function will be the primary task for the future.

### 5.5. CSTP to SeedLink Conversion Performance Test

To test the real-time data stream forwarding latency shown in Figure 10 of Section 5.2, which refers to the latency of packets from entering SharpCEEWPServer, being forwarded to RingServer, and eventually received by slinktool, as well as its concurrency capabilities, we used the dataset described in Section 5.3, along with the modified SharpCEEWPSender, SharpCEEWPServer, and slinktool to simulate scenarios with 200 to 1000 stations. SharpCEEWPSender and SharpCEEWPServer were deployed on the Asus Zenbook laptop mentioned in Section 5.3, using the local loopback address for communication. RingServer and slinktool were deployed on an Ubuntu virtual machine equipped with a 12-core CPU and 32 GB of RAM, also using the local loopback for communication, which is a possible deployment configuration in actual work. The two computers were connected via a local area network and synchronized using the same NTP server.

Before sending the data packets, SharpCEEWPSender modifies their start time to match the transmission time. Since the log and latency calculation accuracy provided by RingServer and slinktool is only at the level of seconds or 0.1 s, we modified slinktool to log data reception times with microsecond precision. The local loopback address transmits data at the operating system kernel level, and the resulting latency is typically less than 1 ms, which can be neglected. Therefore, by subtracting the packet’s start time from the reception time recorded by slinktool, we can calculate the cumulative transmission latency from SharpCEEWPServer receiving the packet, processing and forwarding it, through the local area network to RingServer, and from RingServer to slinktool.

**Figure 16:**
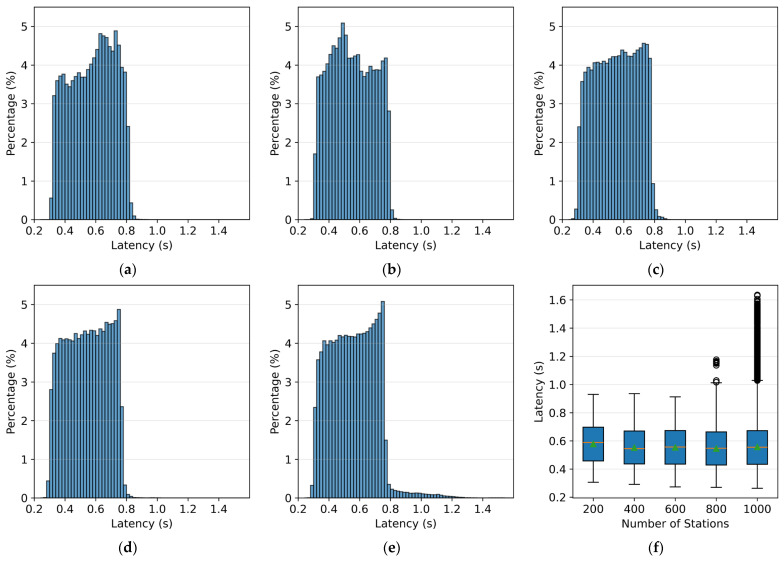
Forwarding system latency: (**a**–**e**) Latency distribution for 200, 400, 600, 800, and 1000 stations; (**f**) Forwarding latency box plot for different station counts, showing the distribution of latencies with median, interquartile range, and outliers. The green triangle represents the mean latency.

In Figure 16a–e, the bin width of the histograms is 0.02 s. For 200 to 600 stations, the transmission latency of the forwarding system falls within the range of 0.25 to 0.9 s. At 800 stations, anomalous values greater than 0.9 s begin to appear. At 1000 stations, the number of anomalies greater than 0.9 s increases significantly. From the box plot in Figure 16f, it can be seen that the average and median latencies are between 0.55 and 0.6 s. Transmission logs recorded by SharpCEEWPServer indicate that the majority of packets experience a latency of about 16 ms from reception to forwarding to RingServer, with the number of packets decreasing rapidly in steps of 32 ms, 48 ms, and 64 ms. This phenomenon suggests that the forwarding process is dominated by a periodic heartbeat mechanism with a 16 ms interval.

In summary, this forwarding system exhibits a latency range of 0.25 to 0.9 s even under low load conditions, and the latency is nearly evenly distributed. Even if the minimum latency is attributed to clock synchronization errors, some packet forwarding latencies still exceed 0.5 s. When the number of stations exceeds 800, there is a noticeable increase in latency anomalies, which worsen as the number of stations increases, indicating the approach of the concurrency performance bottleneck. Therefore, the forwarding system described in Section 5.2 can meet the real-time forwarding needs for approximately 800 stations in terms of concurrency performance. However, the latency amplification limits its applicability to scenarios with higher tolerance, such as temporary networks or routine seismic monitoring, and it cannot meet the requirements for earthquake early warning.

## 6. Conclusions and Discussion, Limitations, and Future Work

This study designed and implemented a lightweight server software based on the real-time communication protocol CSTP used in the China Earthquake Early Warning System. The software adopts a three-layer architecture—Communication/Persistence Layer, Presenter Layer, and View Layer—enabling deep decoupling of functional modules. It also includes performance optimizations tailored for the characteristics of real-time seismic data streams and high-concurrency scenarios, using batch processing, delayed updates, and virtualization display techniques to develop a high-performance waveform display control.

1. Software Test Results Analysis
  (1) Hardware Compatibility and Data Reliability

The hardware compatibility tests showed that all CSTP-compatible seismometers and seismic digitizers were able to complete the registration process with the server and transmit real-time data streams (though other extended features of the protocol were not widely supported by manufacturers). During the 14-h data forwarding test, MiniSEED packets archived by SharpCEEWPServer, RingServer, and Slinktool were consistent, with no packet loss, confirming the feasibility and stability of using SharpCEEWPServer and RingServer as conversion nodes to transform CSTP real-time data streams into SeedLink real-time data streams.

  (2) Concurrency Performance Limits

In the concurrency performance tests, the server successfully handled real-time data streams from 200 to 2000 virtual stations. The server showed low resource consumption and average processing latency when subjected to the shock of an M7.0 earthquake. However, at 1200 stations, the R95 latency increased sharply in a nonlinear fashion, and at 1400 stations, a long-tail effect emerged. This long-tail effect persisted through the end of the test with 2000 stations. Based on the testing environment (laptop-class hardware), the server’s capacity saturated at approximately 1000 three-component seismic stations. When compared with large-scale arrays (e.g., USArray with 400 stations [35], CHINArray-I with 350 [36], CHINArray-II with 660 stations [37]), the performance is sufficient to meet the application requirements.

  (3) Network Robustness and Forwarding Latency

In the network impairment tests, the server was able to quickly recover from network anomalies. Transmission latencies, packet loss, and out-of-order packets in mobile networks affected transmission and processing latency but did not result in data loss. However, network disconnections lasting more than 3 s caused packet loss at the application layer. In the full system forwarding performance test from CSTP to SeedLink, some packet forwarding latencies may exceed 0.5 s, with anomalies appearing when the number of stations exceeded 800. Therefore, the forwarding system based on SharpCEEWPServer currently only meets the needs of scenarios with a higher tolerance for latency, such as earthquake emergency response and seismic array systems.

2. Software Application Value and Future Plans

The lightweight server developed in this study can fully meet the real-time data stream reception needs of temporary seismic networks in both functionality and performance. Additionally, by leveraging RingServer, the server achieves stable conversion from CSTP real-time data streams to SeedLink real-time data streams, seamlessly integrating new seismic instruments into existing earthquake data processing systems, thus offering significant engineering application value. Optimizing transmission latency and implementing packet retransmission functionality are the primary tasks for the future. The current graphical version is only compatible with Windows. Future updates will focus on adopting a cross-platform graphical framework to enable Linux compatibility. Further plans include expanding support for NetSeis/IP, SeedLink, and CD-1 protocol clients, as well as developing a simplified SeedLink server to improve compatibility and coverage across different scenarios, and reduce conversion latency.

## Figures and Tables

**Table 1:** Feature-level CSTP compatibility of the tested instruments.

Feature/Model	Registration	CW ^1^	TW ^2^	TNW ^3^	RC ^4^
TDE-324FI	Y	Y	Y	Y	P
GL-P2B	Y	Y	Y	N	N
GL-PCS120	Y	Y	Y	N	N
HG-D6	Y	Y	N	N	D

^1^ CW: continuous waveform mode. ^2^ TW: trigger with waveform mode. ^3^ TNW: trigger without waveform mode. ^4^ RC: Remote control. Y: supported; P: partially supported; N: not supported; D: device disconnects after receiving control commands.

**Table 2:** Results of Network Impairment Testing.

Metric	Normal	100 ms Latency 5% Packet Loss 10% Out-of-Order	10 Temporary Disconnections (Each ≥ 3 s)
Packets Sent	1425	1425	1425
Packets Received	1425	1425	1387
Integrity Rate (%)	100	100	97.3
R95 Network Latency (ms)	110.76	651.91	114.06
R95 Processing Latency (ms)	0.29	0.52	0.33
R95 End-to-End Latency (ms)	110.98	652.14	114.25

## Data Availability

Restrictions apply to the CSTP protocol draft specification and source code. These materials are internal working documents for a not-yet-public communication standard that underpins the Chinese national Earthquake Early Warning system. Requests for access should be directed to the Guangdong Earthquake Monitoring Center. Restrictions apply to the waveform dataset of the M7.0 earthquake. It was obtained from the China Earthquake Networks Center (CENC). Similar waveform archives are available from the China Earthquake Administration via its Earthquake Science Data Center data-sharing portal (http://esdc.ac.cn/). Other datasets supporting the conclusions of this article and the compiled programs are available from the authors on request for research and non-commercial seismological use.

## Abbreviations

The following abbreviations are used in this manuscript:

LISS	Live Internet Seismic Server
CEA	China Earthquake Administration
EEW	Earthquake early warning
EEWS	Earthquake early warning system
FIFO	first-in–first-out
